# A patient with paroxysmal nocturnal hemoglobinuria being treated with eculizumab who underwent laparoscopic cholecystectomy: report of a case

**DOI:** 10.1186/s40792-015-0059-8

**Published:** 2015-07-11

**Authors:** Makoto Moriyama, Takuya Nagata, Isaku Yoshioka, Isaya Hashimoto, Koshi Matsui, Tomoyuki Okumura, Kazuhiro Tsukada

**Affiliations:** Department of Surgery & Science, University of Toyama, 2630 Sugitani, Toyama-city, Toyama 930-0194 Japan

**Keywords:** Paroxysmal nocturnal hemoglobinuria, Eculizumab, Laparoscopic cholecystectomy

## Abstract

Paroxysmal nocturnal hemoglobinuria (PNH) is acquired hemolytic anemia characterized by symptoms such as anemia and hemoglobinuria. In recent years, eculizumab as an anti-complement (C5) monoclonal antibody has been used for PNH and shown to have marked effects. We performed laparoscopic cholecystectomy in a patient with PNH being treated with eculizumab, and could avoid the risk of perioperative hemolysis and thrombosis. [Patient] The patient was a 48-year-old female who had developed PNH when she was 39 years old. At the age of 46 years, eculizumab administration was initiated once every 2 weeks. During the administration period, neither the progression of anemia nor hemoglobinuria was observed. In March 2013, gallstones were detected, and she was referred to our hospital for surgery. Eculizumab was administered 10 days before surgery, and laparoscopic cholecystectomy was performed in May 2013. After the operation, for the prevention of thrombosis, elastic stockings and a foot pump were used without anticoagulant administration. After the operation, neither the progression of anemia nor hemoglobinuria was observed. On postoperative day 5, eculizumab was administered as planned, and she showed a favorable general condition and was discharged. [Discussion] Perioperative care in PNH patients was conventionally considered to involve a high risk of developing anemia, thrombosis, or infection. However, after the advent of eculizumab, the control of the symptoms of PNH became possible in many patients. In this patient with PNH being treated with eculizumab, safe perioperative management was possible without the development of complications.

## Background

Paroxysmal nocturnal hemoglobinuria (PNH) is characterized by the appearance of complement-sensitive erythrocytes due to an acquired mutation in hematopoietic cells and resulting chronic intravascular hemolysis [1, 2]. PNH affects 6.9 per million people. As perioperative care for PNH, the prevention of hemolytic attacks and thrombosis is important. The mechanism of hemolytic attacks is considered to be complement activation due to perioperative invasion, acidosis, and hypoxemia. For the prevention of thrombosis, since hemolysis increases the activity of the coagulation system, the use of drugs such as heparin is recommended [3]. In recent years, the administration of eculizumab as an anti-complement (C5) monoclonal antibody for PNH has produced marked effects.

We report a patient with PNH being treated with eculizumab in whom laparoscopic cholecystectomy was performed, and the risk of perioperative hemolysis or thrombosis could be avoided.

## Case presentation

### Patient

A 48-year-old female.

### Chief complaint

No symptoms.

### Past history

She had developed PNH when she was 39 years old. At the age of 46 years, eculizumab administration once every 2 weeks was initiated, and neither progression of anemia nor hemoglobinuria was observed during the administration period.

### Present illness

In March 2013, she visited a local hospital due to jaundice and abdominal pain, and was diagnosed with cholecystolithiasis and choledocholithiasis. She continued administration of eculizumab once every 2 weeks then for two years. She was referred to the Department of Gastroenterology. Common bile duct stones were removed using endoscopic sphinterotomy (EST), and she was then referred to our department for surgery for cholecystolithiasis.

### Physical findings

She was 165.3 cm tall and weighed 74.7 kg, and showed a BMI of 27.94, blood pressure of 127/94 mmHg, pulse of 74 bpm, body temperature of 36.8°C, and no anemia or jaundice in the conjunctiva.

### Blood examination findings

The leukocyte count was 2,930, and the Hb value was 8.2, both showing decreases.

### ERCP findings

**Fig. 1:**
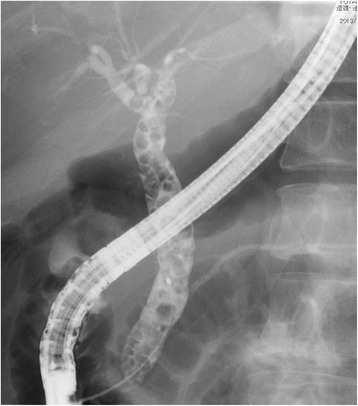
There were many stones accumulating in the common bile duct

Radiolucent images in the common bile duct showing aggregation and extension to the porta hepatis were observed, suggesting many stones (Fig. 1).

### CT findings

**Fig. 2:**
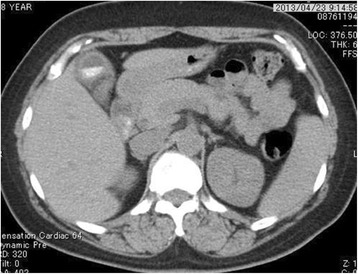
High-density shadows were observed, suggesting stones in the gallbladder

There were many high-density shadows suggesting gallstones (Fig. 2).

### MRCP findings

**Fig. 3:**
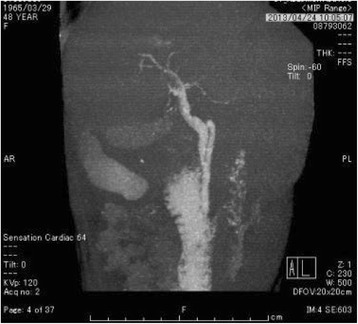
After EST, no stones were observed in the common bile duct. There were numerous stones in the gallbladder

After EST, there were no findings suggesting stones in the common bile duct. In the gallbladder, many stones were observed (Fig. 3).

### Surgical findings

Eculizumab was administered 10 days before surgery, and laparoscopic cholecystectomy was performed. The operation time was 1 hour and 59 minutes, and the bleeding volume was small. No complications occurred during the operation.

### Postoperative course

**Fig. 4:**
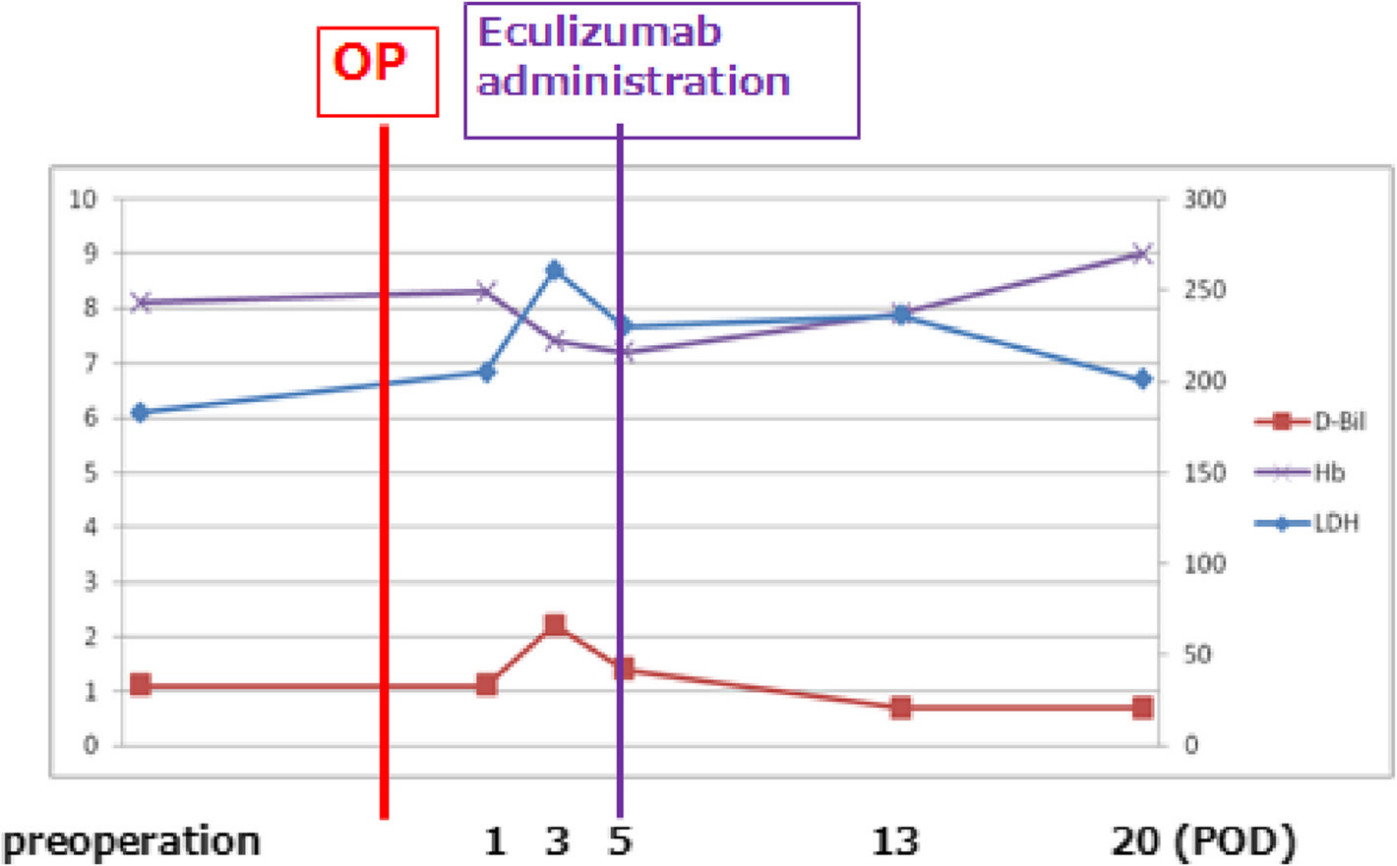
After the operation, bilirubin and LDH slightly increased but showed rapid improvement. No progression of anemia requiring blood transfusion was observed

For the prevention of thrombosis, elastic stockings and a foot pump were used without anticoagulant administration. After the operation, neither the progression of anemia or hemoglobinuria was observed. Slight increases in D-Bil and LDH with peaks on postoperative day 3 and a slight decrease in Hb were observed, but rapidly improved (Fig. 4). On postoperative day 5, after eculizumab had been administered as planned, she showed a favorable general condition, and was discharged.

## Discussion

PNH is acquired hemolytic anemia characterized by intravascular hemolysis caused by increased complement sensitivity due to the deficiency of complement regulatory factor on the cell membrane surface [4]. This disease is rare, affecting 6.9 per million people, and slightly more frequent in males (male : female ratio = 1 : 0.77) [5].

**Fig. 5:**
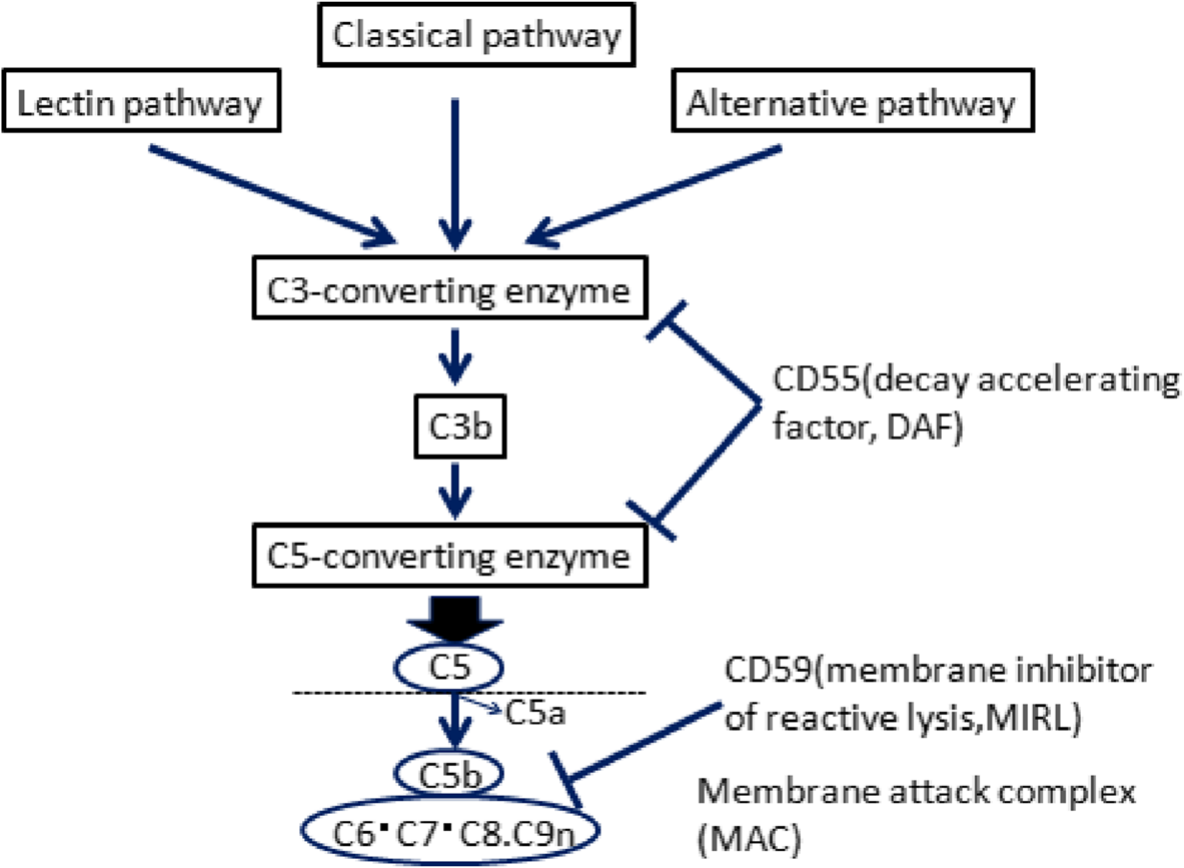
Eculizumab acts on C5 in the complement cascade, blocking complement activation by the C5-converting enzyme

Eculizumab as a treatment for PNH is an anti-complement (C5) monoclonal antibody, and acts on C5 in the complement cascade, blocking complement activation by the C5-converting enzyme and inhibiting hemolytic attacks (Fig. 5) [6, 7]. In Japan, this drug was approved as a treatment for PNH in 2010. As perioperative care in PNH patients, the prevention of hemolytic attacks due to surgical invasion and thrombosis associated with increased activity in the coagulation system is important. Conventionally, the pre- and postoperative administration of heparin calcium or dalteparin sodium was recommended as perioperative care [8, 9]. To prevent hemolytic attacks, the use of steroids was also recommended [10]. However, after the advent of eculizumab, patients in whom PNH symptoms can be controlled with this drug have been increasing. In our patient, hemolytic attacks and hemolysis could be avoided during the perioperative period without using anticoagulants or steroids.

Eculizumab is administerd once in two weeks, but the blood concentration two weeks after administration eculizumab 900 mg is approximately 110 μg/ml, and blood concentration more than 35 μg/ml is recommended. Therefore, as for the timing of the operation, it is thought that it is no problem when if during a administeration period of eculizimab.

So far, patients who underwent cholecystectomy in PNH patients under eculizumab administration has been reported two cases, both of which are open cholecystectomy. The patient who underwent laparoscopic-cholecystectomy in PNH patients under eculizumab administration has been slightly observed in the conference proceedings. In any case, it has not happened serious complications in the perioperative in PNH patients under eculizumab administration [11, 12].

Surgically treated PNH patients are extremely rare. In this patient, we had difficulty in determining the extent of preventive measures, such as the use of coagulants or steroids. However, since steroid administration was previously effective against hemolytic attacks in this patient, we prepared for rapid steroid administration and blood transfusion against the possible development of hemolytic attacks. However, neither hemolytic attacks nor thrombosis developed. These results suggest that eculizumab adequately acted on the complement system even during the perioperative period, enabling safe perioperative care without severe complications in this patient who underwent laparoscopic cholecystectomy as minimally invasive surgery.

## Conclusion

We encountered a patient with PNH being treated with eculizumab in whom safe perioperative care was possible without complications.

## Consent

Written informed consent was obtained from the patient for publication of this Case report and any accompanying images.
